# Assessing Competence of Broccoli Consumption on Inflammatory and Antioxidant Pathways in Restraint-Induced Models: Estimation in Rat Hippocampus and Prefrontal Cortex

**DOI:** 10.1155/2013/590379

**Published:** 2013-07-11

**Authors:** Leila Khalaj, Sara Chavoshi Nejad, Marzieh Mohammadi, Sadaf Sarraf Zadeh, Marieh Hossein Pour, Ghorbangol Ashabi, Fariba Khodagholi, Abolhassan Ahmadiani

**Affiliations:** ^1^Physiology and Pharmacology Department, Medical School, Alborz University of Medical Sciences, Eshteraki Avenue, Baghestan Boulevard, P.O. Box 3148/561 Karaj, Iran; ^2^Neuroscience Research Center, Shahid Beheshti University of Medical Sciences, Tehran, Iran; ^3^Department of Pharmacology, Medical Faculty, University Malaya, Kuala Lumpur, Malaysia

## Abstract

A growing body of evidence advocated the protective and therapeutic potential of natural compounds and phytochemicals used in diets against pathological conditions. Herein, the outcome of dietary whole broccoli consumption prior to restraint stress has been investigated in the hippocampus and prefrontal cortex of male rats, two important regions involved in the processing of responses to stressful events. Interestingly, a region-specific effect was detected regarding some of antioxidant defense system factors: nuclear factor erythroid-derived 2-related factor 2 (Nrf-2) antioxidant pathway, mitochondrial prosurvival proteins involved in mitochondrial biogenesis, and apoptotic cell death proteins. Dietary broccoli supplementation modulated the restraint-induced changes towards a consistent overall protection in the hippocampus. In the prefrontal cortex, however, despite activation of most of the protective factors, presumably as an attempt to save the system against the stress insult, some detrimental outcomes such as induced malate dehydrogenase (MDA) level and cleaved form of caspase-3 were detectable. Such diversity may be attributed in one hand to the different basic levels and/or availability of defensive mechanisms within the two studied cerebral regions, and on the other hand to the probable dose-dependent and hormetic effects of whole broccoli. More experiments are essential to demonstrate these assumptions.

## 1. Introduction

Epidemiological studies have revealed that diets rich in plant-derived foods have protective effects on human health, and the impact of dietary factors on health and longevity is increasingly appreciated [1]. Regular dietary consumption of vegetables and fruits containing phytochemicals from childhood to adulthood are associated with reduced risk of several major pathological conditions, including cancer, inflammatory, and cardiovascular, as well as neurotraumatic (stroke, traumatic brain injury, and spinal cord injury), neurodegenerative (Alzheimer's disease (AD), Parkinson's disease (PD), and cataracts) and neuropsychiatric diseases (depression, Schizophrenia, and bipolar disorders). Phytochemicals derived from plants also form the backbone of traditional medicine, which uses plant preparations as a source of drugs [2].

One of the families rich in phytochemicals is Cruciferous vegetables, including Brussels sprouts, Cauliflower, Cabbage, and Broccoli [3]. Broccoli contains many bioactive compounds, such as vitamins C and E, quercetin, kaempferol glycosides, and several glucosinolates including glucoraphanin. A key bioactive component suggested to be responsible for much of the activities of broccoli is an isothiocyanate called sulforaphane, which is in fact a hydrolysis product of glucoraphanin [4].

There are a large number of studies in support of cytoprotective properties of sulforaphane in several in vivo experimental paradigms associated with oxidative stress such as focal cerebral ischemia, brain inflammation, intracerebral hemorrhage, ischemia-reperfusion (I/R) induced acute renal failure, streptozocin-induced diabetes, hepatotoxicity, and cardiac I/R, as well as in various in vitro studies [5]. However, it should be noticed that not all the protective effects of broccoli are attributed to sulforaphane. Because other bioactive compounds in broccoli such as kaempferol have also proved a wide range of pharmacological activities, including antioxidant, anti-inflammatory, antimicrobial, and antidiabetic [6]. It is not certain but it seems reasonable to suggest that presence of other components besides sulforaphane in broccoli, when it is consumed as a whole in diets, may add to its health benefits. In the study by Keck et al., it is reported that broccoli caused a significant increase in hepatic ethoxyresorufin O-deethylase (EROD: a detoxification enzyme; 36.5%), whereas sulforaphane caused a slight (9%) but not significant decrease in EROD. Improved bioactivity of whole broccoli in this study is attributed to its additional bioactive components [7].

Studies which focus on examining the properties of the whole broccoli in different experimental contexts are in fact more relevant and have more interests regarding the human diets and lifestyle. It has been reported that dried broccoli sprouts were able to attenuate oxidative stress, hypertension, and inflammation in stroke-prone spontaneously hypertensive rats [8]. A cardioprotective effect was found after feeding broccoli for 30 days to rats in isolated heart preparation submitted to ischemia and reperfusion (I/R), via strengthening of redox signaling by overexpressing nuclear factor erythroid-derived 2-related factor 2 (Nrf-2) and heme oxygenase-1 (HO-1) [9]. In the same context, superior cardioprotective effect of the steamed over cooked broccoli has been documented, because of the ability of fresh broccoli to perform redox signaling of thioredoxin [10]. Dietary administration of broccoli seeds has shown effectiveness at inducing antioxidant and detoxification proteins, both in vivo, in stomach, small intestine, and liver of wild-type mice, and ex vivo, in an Nrf-2-dependent manner [11]. Broccoli has been able to slow or prevent colon cancer as well [12]. In the rats fed broccoli sprouts, oxidative damage in the brain was decreased, as were other signs of inflammation, shown by fewer activated macrophages or other monocytes in CNS tissue, and decreases in iNOS levels [13]. To the best of our knowledge, it appears that there are very limited studies examining the beneficial potential of whole broccoli consumption against various pathological contexts, and there is even a lack of evidence regarding the effects of whole broccoli consumption against neuropathological conditions.

Encountering different types of stresses is inevitable during human lives. Various brain regions contribute to the regulation of the important components of stress response. Areas such as amygdale, prefrontal cortex, and hippocampus are of particular importance [14–16]. Such areas are also compromised in neurodegenerative diseases including AD, PD, and Huntington's disease (HD). In AD, neurodegeneration occurs mostly in the hippocampus and amygdale [17], in PD, neurons in the substantia nigra and striatum are affected by disease [18], whereas in HD, neuronal populations in the striatum, globus pallidus, and frontal cortex are degenerated [19]. Stress and neurodegenerative diseases also share common pathological changes in response to insults, including ROS production [20], inflammation [21], and mitochondrial dysfunction [22], as well as apoptotic cell death pathways [23].

Stress is in fact considered to predispose the brain to various neuropathological conditions [14, 15, 24]. Physical and psychological stresses are believed to be associated with a broad range of diseases, such as mental disorders, anxiety, and depression [25], as well as neurodegenerative diseases [26, 27]. According to a growing body of evidence, psychological stress is implicated as a potential contributing factor to the development of AD [26, 28–31].

Using the restraint or immobilization induced stress is one of the most common stress models in experimental studies [16]. While stressing a rat with restraint has a physical component, it acts primarily as a psychological stressor [32]. Using this approach in the present study, we aimed to examine the beneficial potential of whole broccoli consumption, which is the most common route of broccoli consumption in human diets, against stress-induced changes in the neuronal context. We specifically focused on the hippocampus and cortex, two important cerebral regions involved in the processing of stress responses, and the important molecular components contributing in the pathophysiology of both stress and neurodegenerative disorders, including antioxidant defense system, antioxidant Nrf-2 signaling pathway, mitochondrial prosurvival factors involved in the mitochondrial biogenesis, and inflammatory as well as apoptotic cell death factors. Such assessments may provide some insights regarding the preventive/therapeutic potential of dietary broccoli consumption against pathological conditions of CNS which possess a stress component.

## 2. Materials and Methods

### 2.1. Animals

Male Wistar rats (275–300 g) were housed in standard cages under controlled temperature (22 ± 2°C), humidity, and a 12 hr light/dark cycle (light on 07:00–19:00), with food and water provided *ad libitum*. Rats acclimated to the facilities for 1 week and then were randomly assigned to the experimental groups, with control, stressed, and broccoli pretreated rats, housed in separate chambers. Experimentation was approved by the Ethics Committee of Shahid Beheshti Medical University in accordance with the international guidelines for animal experiments. All efforts were made to minimize animal suffering and to reduce the number of animals used.

### 2.2. Experimental Groups

Fresh broccoli, provided from local supermarket, was blanched by subjecting it to steaming, and then a slurry was made with adding water in a ratio of 1 : 3*·*5 (w/w) and mixing in a blender, according to McWalter et al. [10]. The rats were randomly assigned to one of the three groups: (1) control, (2) stress, and (3) treatment (broccoli + stress), receiving the steamed whole broccoli in diet as pretreatment (*n* = 12/group). Animals were fed *ad libitum* regular rat chow with free access to water. The broccoli-treated rats were fed with 1 mL broccoli slurry (1.5 g/kg body weight per day) for 10 days, while the stress group of rats was gavaged 1 mL water for the same period of time. One hour after the final dose, animals in the stress and treatment groups were subjected to 6 hours of restraint stress. Additional animals were utilized to assess NF-*κ*B, Nrf-2, and AIF proteins in the nuclear fraction of each experimental group (*n* = 6). An extra number of animals were also specified to receive “broccoli only” treatment (*n* = 6).

### 2.3. Restraint Stress

Rats were exposed to stress between 09:00 and 15:00 in the animal homeroom. The restraint was performed using tightly fitted rodent restrainers for 6 hours [33, 34] in their home cages. Control animals were not subjected to stress but were handled at 9:00. Animals were euthanized immediately after restraint (still in the restrainer) using sodium pentobarbital. Blood for plasma determinations was collected by cardiac puncture and anticoagulated in the presence of trisodium citrate (3.15% w:v, 1 voL citrate per 9 vol blood). After decapitations, the brain was removed from the skull and the hippocampus and prefrontal cortex structures were excised from the brain and kept at −80°C to be used for western blot (*n* = 6/group) and biochemical assessments (*n* = 6/group).

### 2.4. Plasma Corticosterone Level

Plasma was extracted within 1 hr of collecting blood samples by centrifuging the samples at 1000 g for 15 min immediately after stress. All plasma samples were kept at −80°C before assay by using a commercially available corticosterone ELISA kit (Cayman Chemical, USA).

### 2.5. Biochemical Analyses

Frozen hippocampus and prefrontal cortex samples were weighed and homogenized in 100 mmol/L phosphate buffer (pH 7.4) containing 0.05% sodium azide in an ice bath. The homogenate was sonicated for 30 s and centrifuged (5,000 g for 10 min). The supernatant was frozen at −80°C in aliquots until further use. The protein content of the supernatant was determined using the Bradford method [35].

#### 2.5.1. Measurement of Lipid Peroxidation

The MDA level as an index of lipid peroxidation was measured by the double heating method [36]. The method is based on spectrophotometric measurement of the purple color generated by the reaction of thiobarbituric acid (TBA) with MDA. A 0.5 mL of hippocampal homogenate was mixed with 2.5 mL of Trichloroacetic acid (TCA) (10% w/v) solution followed by boiling in a water bath for 15 min. After cooling to room temperature, the samples were centrifuged at 1,500 g for 10 min and 2 mL of each sample supernatant was transferred to a test tube containing 1 mL of TBA solution (0.67% w/v). Each tube was then placed in boiling water for 15 min. After cooling to room temperature, the absorbance was measured at 532 nm with respect to the blank solution. The concentration of MDA was calculated based on the absorbance coefficient of the TBA-MDA complex (e = 1.56 9 105 cm^−1^ M^−1^) and expressed in nmol/mg protein.

#### 2.5.2. Superoxide Dismutase Activity Assay

SOD activity was measured based on the extent of inhibition of amino blue tetrazolium formazan formation in the mixture of nicotinamide adenine dinucleotide (NADH), phenazine methosulphate (PMS), and nitroblue tetrazolium (NBT) [37]. Assay mixture contained 0.1 mL of supernatant, 1.2 mL of sodium pyrophosphate buffer (pH 8.3, 0.052 M), 0.1 mL of phenazine methosulphate (186 lM), 0.3 mL of nitroblue tetrazolium (300 lM), and 0.2 mL of NADH (750 lM). The reaction was started by addition of 0.1 mL of NADH. After incubation at 30°C for 90 s, the reaction was stopped by addition of 0.1 mL of glacial acetic acid. The reaction mixture was stirred vigorously with 4.0 mL of n-butanol. Color intensity of the chromogen in butanol was measured spectrophotometrically at 560 nm. One unit of enzyme activity was defined as the amount of enzyme which caused 50% inhibition of NBT reduction per mg of protein.

#### 2.5.3. Measurement of Gluthatione Levels

The concentration of GSH was determined in whole tissue supernatant using dithionitrobenzoic acid (DTNB) method at 412 nm [38].

### 2.6. Preparation of Total Protein Extracts

The hippocampus and prefrontal cortex were dissected on ice in ice-cold 125 mmol/L Tris-HCl, pH 7.4, containing 320 mmol/L sucrose, 2 mmol/L sodium orthovanadate, 20 mmol/L sodium diphosphate decahydrate, 20 mmol/L DL-a-glycerophosphate, 0.1 mmol/L phenylmethylsulfonyl fluoride, and 5 mg/mL each of antipain, aprotinin, and leupeptin (homogenization buffer). Total protein extract was collected by centrifugation at 13000 g for 5 min. The samples were stored at −80°C until needed for western blot analysis [39].

### 2.7. Preparation of Nuclear Protein Extracts for Assessment of Nuclear Proteins

Tissues were homogenized with 300 mL lysis buffer (10 mmol/L N-2-hydroxyethylpiperazine-N-2-ethanesulfonic acid (pH 7.9), 1 mmol/L EDTA, 1 mmol/L EGTA, 10 mmol/L KCl, 1 mmol/L dithiothreitol, 0.5 mmol/L phenylmethylsulfonyl fluoride, 0.1 mg/mL aprotinin, 1 mg/mL leupeptin, 1 mg/mL Na-p-tosyl-L-lysine-chloromethyl ketone, 5 mmol/L NaF, 1 mmol/L NaVO_4_, 0.5 mmol/L sucrose, and 10 mmol/L Na_2_MoO_4_). After 15 min, Nonidet P-40 (Roche, Mannheim, Germany) was added to reach a concentration of 0.5%. The tubes were gently vortexed for 15 sec, and nuclei were collected by centrifugation at 8000 g for 5 min. The pellets were resuspended in 100 ml buffer supplemented with 20% glycerol and 0.4 mol/L KCl and gently shaken for 30 min at 4°C. Nuclear protein extracts were obtained by centrifugation at 13,000 g for 5 min, and aliquots of the supernatant were stored at −80°C. All steps were carried out at 4°C [40].

### 2.8. Western Blotting

Western blotting was used to measure the protein expression of PGC-1*α* (ABCAM; 1 *µ*g/mL), NRF-1 (Santa Cruz; 1/1000), TFAM (BioVision; 0.5 *μ*g/mL), cleaved caspase-3 and AIF (Cell Signaling Technology; 1/1000), TNF-*α* (ABCAM; 1/2000), NF-*κ*B p65 subunit (Cell Signaling Technology; 1/2000), Nrf-2 (ABCAM; 1 *μ*g/mL), HO-1 (Cell Signaling Technology; 1/1000), and NQO-1 (Santa Cruz; 1/200).

Standard plots were generated using bovine serum albumin. Lysates equivalent to 30 *μ*g of protein were resolved on SDS—10% polyacrylamide gel electrophoresis, and transferred to Nitrocellulose membrane (Porablot, Macherey-Nagel, Germany). Then blots were blocked in 2% ECL advanced kit blocking reagent (Amersham Biosciences) and probed with primary antibodies overnight at 4°C. After washing, membranes were incubated for 90 min at room temperature with horseradish peroxidase-conjugated secondary antibodies as follows: rabbit and mouse IgG-HRP-linked antibodies (Cell Signaling; 1/10000). Blots were revealed by ECL advanced kit (Amersham Biosciences). To normalize for protein content, blots were stripped in stripping buffer containing 100 mM 2-mercaptoethanol, 2% (w/v) SDS, 62.5 mM Tris-HCl (pH = 6.7) and then probed with anti-*β*-actin and anti-lamin B2 antibodies (Santa Cruz Biotechnology; 0.5 *μ*g/mL and 1/1000, resp.). The density of bands was quantified using NIH Image J, and the ratio to *β*-actin or lamin B2 was calculated.

### 2.9. Statistical Analysis

All the western blot, biochemical, and plasma corticosterone level assessment data were analyzed by a one-way analysis of variance (ANOVA) followed by Tukey HSD for multiple comparisons, using SPSS 16.0 package programs. Data are expressed as mean ± SEM and statistical significance was set at *P* < 0.05.

## 3. Results

### 3.1. Whole Broccoli Consumption Attenuated Restraint Stress-Induced Plasma Corticosterone Level in Rats

As represented in Table 1, the significant induction of plasma corticosterone level in response to 6 hours of restraint-induced stress compared with control group (*P* < 0.05) was reversed in the presence of broccoli, to a significant extent (*P* < 0.05).

### 3.2. Effect of Whole Broccoli Consumption on the Antioxidant Defense System Factors of Hippocampus and Prefrontal Cortex: SOD, GSH, and MDA

**Figure 1:**
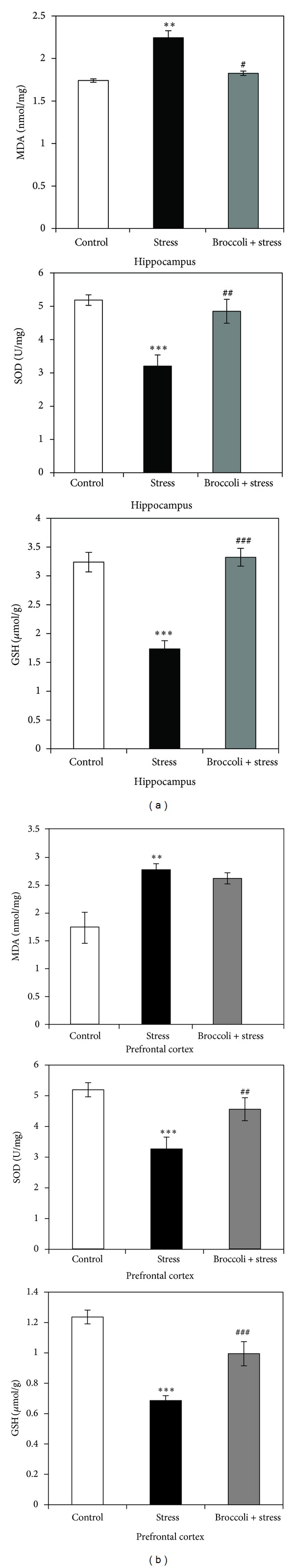
Biochemical assessment of antioxidant defense system in the hippocampus (a) and prefrontal cortex (b) tissues derived after 6 hours of restraint-induced stress in experimental groups (*n* = 6), SOD activity, and GSH and MDA levels; bars indicate the mean ± SEM; one-way ANOVA; **P < 0.01, ***P < 0.01  compared with the control; ^#^ *P* < 0.05  ^##^ *P* < 0.01, ^###^ *P* < 0.001 compared with the stress.

Figure 1 illustrates the data obtained from assessment of antioxidant defense system, using SOD activity and GSH level in one hand and MDA level as the byproduct of lipid peroxidation on the other hand, in both hippocampus and prefrontal cortex. Six hours of restraint-induced stress reduced SOD activity and depleted GSH in hippocampus and prefrontal cortex compared with the control (*P* < 0.001 and *P* < 0.001, resp.). The presence of broccoli increased SOD activity (hippocampus: *P* < 0.01, prefrontal cortex: *P* < 0.01) and GSH level (hippocampus: *P* < 0.001, prefrontal cortex: *P* < 0.001) in response to stress, although to a lesser extent in prefrontal cortex compared with the hippocampus. However, despite the significant reduction of MDA level in the hippocampus by broccoli compared with the stress group (*P* < 0.01), it was not able to reduce MDA level in the prefrontal cortex against stress insult.

### 3.3. Effect of Whole Broccoli Consumption on Mitochondrial Prosurvival Proteins Involved in the Mitochondrial Biogenesis Pathway: PGC-1*α*, NRF-1, and TFAM

**Figure 2:**
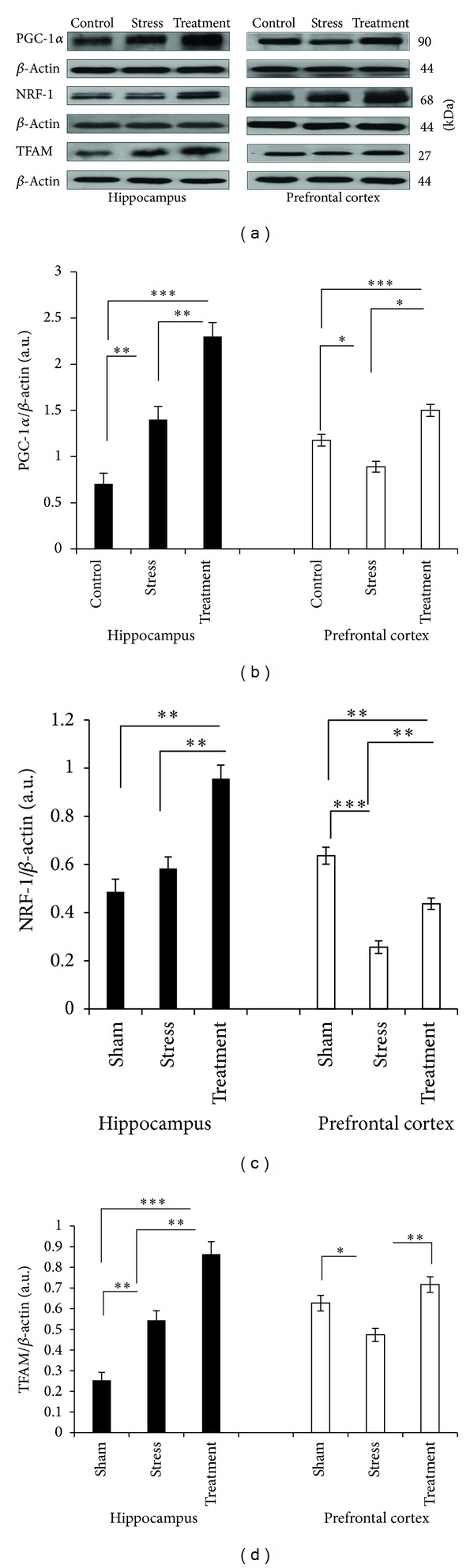
Western blot analysis to measure the expression of major mitochondrial prosurvival proteins involved in the mitochondrial biogenesis, PGC-1*α*, NRF-1, and TFAM, in the hippocampus and prefrontal cortex tissues derived after 6 hours of restraint-induced stress in experimental groups. (a) Immunoblot bands of PGC-1*α*, NRF-1, and TFAM, as well as their relevant *β*-actin bands. ((b)–(d)) The densities of corresponding bands were measured and the ratios to *β*-actin were calculated and represented as arbitrary units on the graphs for each experimental group (*n* = 6). Bars indicate the mean ± SEM; one-way ANOVA; *P < 0.05; **P < 0.01; ***P < 0.001.

As shown in Figure 2, in the hippocampus stress, insult increased PGC-1*α* and TFAM protein levels (*P* < 0.01, *P* < 0.01, resp.), while the amount of NRF-1 showed a nonsignificant incline in comparison with the control state. Broccoli pretreatment caused a significant induction of all three mitochondrial prosurvival factors compared with both stress and control groups.

In prefrontal cortex, while, in response to stress insult, the amount of PGC-1*α*, NRF-1, and TFAM decreased considerably compared with control (*P* < 0.05, *P* < 0.001, *P* < 0.05, and resp.), broccoli was able to reverse such pattern, as in its presence the level of all factors showed a significant incline in comparison with the stressed status (*P* < 0.001, *P* < 0.01, and *P* < 0.01, resp.). 

### 3.4. Effect of Whole Broccoli Consumption on Some Proteins of Nrf-2 Antioxidant Pathway: Nrf-2, HO-1, and NQO-1

**Figure 3:**
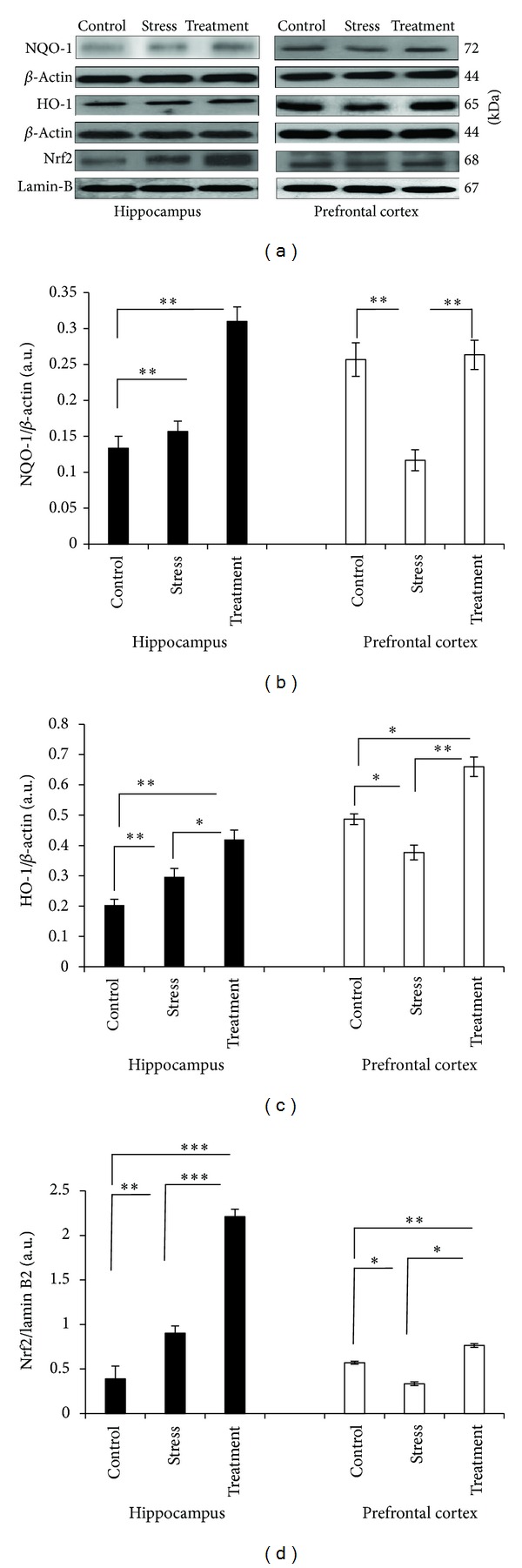
Western blot analysis to measure the expression of the major proteins of the antioxidant Nrf-2 pathway, nuclear Nrf-2, HO-1, and NQO-1 in the hippocampus and prefrontal cortex tissues derived after 6 hours of restraint-induced stress in experimental groups. (a) Immunoblot bands of NQO-1, HO-1, and nuclear Nrf-2, as well as their relevant *β*-actin and lamin B2. ((b)–(d)) The densities of corresponding bands were measured and their respective ratios to *β*-actin and lamin B2 and were calculated and represented as arbitrary units on the graphs for each experimental group (*n* = 6). Bars indicate the mean ± SEM; one-way ANOVA; *P < 0.05; **P < 0.01; ***P < 0.001.

The nuclear level of Nrf-2, as well as HO-1 and NQO-1 in the experimental groups of the present study, is shown in Figure 3. While, in the hippocampus, nuclear presence of Nrf-2 and the level of HO-1 were induced in the stressed condition (*P* < 0.01, and *P* < 0.01, resp.), the amount of NQO-1 showed a nonsignificant incline compared with the control group. Interestingly, broccoli consumption induced all three factors in response to stress (Nrf-2: *P* < 0.001, NQO-1: *P* < 0.01, HO-1: *P* < 0.01) compared with the control state.

In prefrontal cortex, the molecular changes of Nrf-2 signaling pathway followed the same pattern of pro-survival factors of mitochondrial biogenesis pathway; being reduced in the stress situation (*P* < 0.05 for Nrf-2 and HO-1, *P* < 0.01 for NQO-1), while induced when broccoli was present against the stress insult (*P* < 0.01 for Nrf-2 and NQO-1, *P* < 0.05 for HO-1).

### 3.5. Effect of Whole Broccoli Consumption on Proteins of Inflammatory Pathway: NF-*κ*B p65 and TNF-*α*

**Figure 4:**
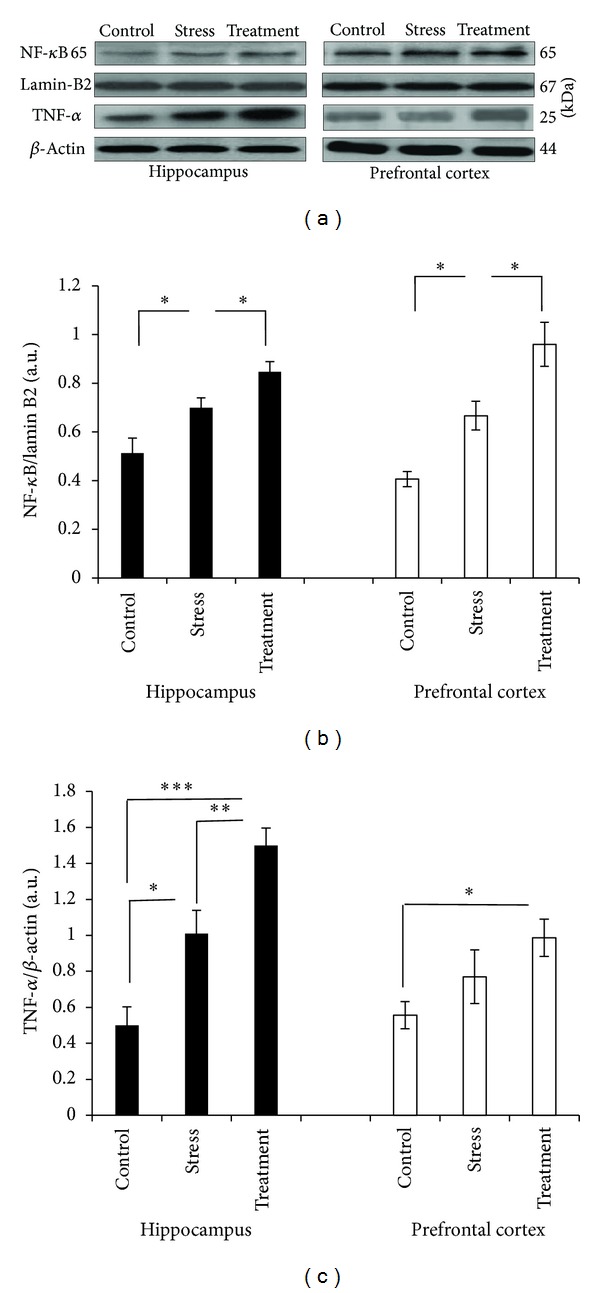
Western blot analysis to measure the expression of inflammatory proteins, nuclear NF-*κ*B p65, and TNF-*α* in the hippocampus and prefrontal cortex tissues derived after 6 hours of restraint-induced stress in experimental groups. (a) Immunoblot bands of nuclear NF-*κ*B p65 and TNF-*α*, as well as their relevant lamin B2 and *β*-actin bands. ((b) and (c)) The densities of corresponding bands were measured and the ratios to lamin B2 and *β*-actin were calculated and represented as arbitrary units on the graphs for each experimental group (*n* = 6). Bars indicate the mean ± SEM; one-way ANOVA; *P < 0.05; **P < 0.01; ***P < 0.001.

As depicted in Figure 4, the restraint stress increased the nuclear presence of NF-*κ*B p65 and the level of TNF-*α* in comparison with the control in both hippocampus (*P* < 0.05, and *P* < 0.05, resp.), and prefrontal cortex (NF-*κ*B p65: *P* < 0.05, TNF-*α*: non-significant). A further incline of their level was detected in the hippocampus (*P* < 0.05, and *P* < 0.01, resp., for NF-*κ*B p65 and TNF-*α*) and prefrontal cortex (NF-*κ*B p65: *P* < 0.05, TNF-*α*: non-significant) of broccoli-receiving animals against the stress insult.

### 3.6. Effect of Whole Broccoli Consumption on Protein Levels of Apoptotic Cell Death Factors: Cleaved Caspase-3 and AIF

**Figure 5:**
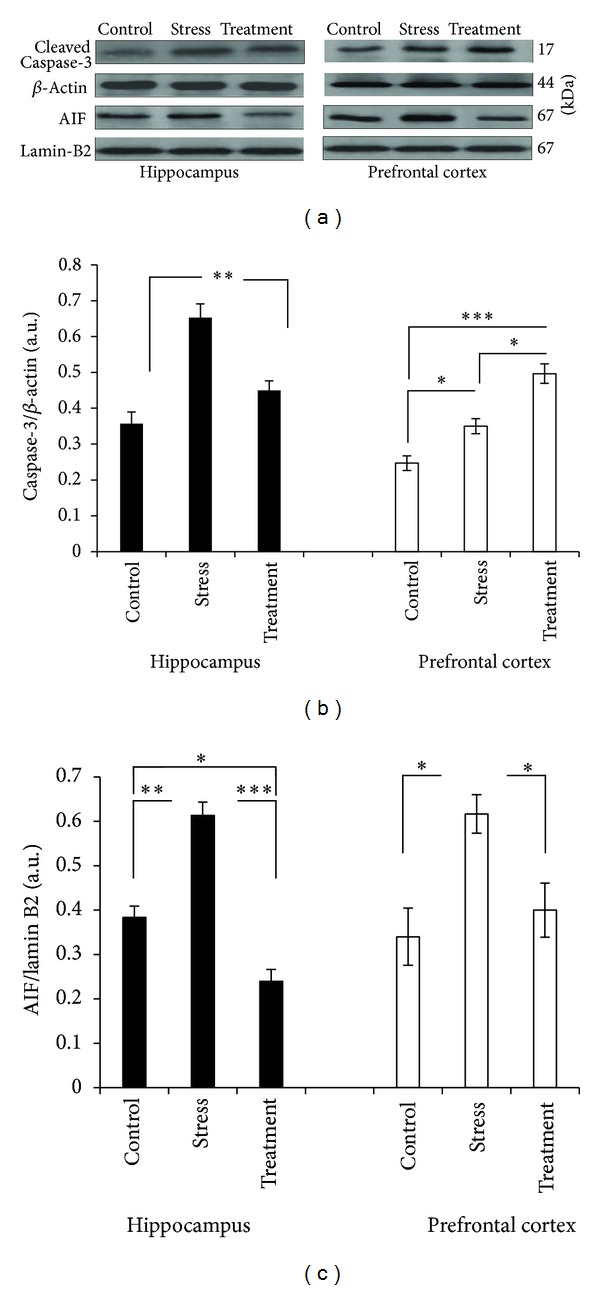
Western blot analysis to measure the expression of the important proteins of the caspase-dependent and caspase-independent apoptotic cell death, cleaved caspase-3 and AIF, respectively, in the hippocampus and prefrontal cortex tissues derived after 6 hours of restraint-induced stress in experimental groups. (a) Immunoblot bands of cleaved caspase-3 and nuclear AIF, as well as their relevant *β*-actin and lamin B2 bands. Changes in the expression of 17 kDa fragment of cleaved caspase-3 are considered. ((b) and (c)) The densities of corresponding bands were measured and the ratios to *β*-actin and lamin B2 were calculated and represented as arbitrary units on the graph for each experimental group (*n* = 6). Bars indicate the mean ± SEM; one-way ANOVA; *P < 0.05; **P < 0.01; ***P < 0.001.

In the hippocampus, activation of caspase-3 and nuclear AIF significantly increased in response to stress (Figure 5, *P* < 0.01, and *P* < 0.01, resp.), but broccoli consumption proved the ability to reduce such deteriorative changes of stressed condition considerably (*P* < 0.01, and *P* < 0.001, resp.). Similarly, in the prefrontal cortex, the stress insult caused a significant incline of these prodeath factors compared with the control (*P* < 0.05 for both). However, in the presence of broccoli, while the amount of nuclear AIF decreased significantly (*P* < 0.05), surprisingly the cleaved form of caspase-3 showed a significant further increase compared with the stress group (*P* < 0.05).

## 4. Discussion 

 In this study, the whole broccoli consumption as pretreatment in male rats proved protection by declining the increased level of plasma corticosterone in response to 6 hours of restraint-induced stress. However, the alternation of biochemical and molecular factors in the hippocampus prefrontal cortex did not follow a similar pattern in response to this equal model and amount of stress, at least at the time point of our analysis.

 Stressful events are believed to increase production of free radicals in the brain and other organs [15, 41, 42]. Evaluation of antioxidant defense system in the present study indicated declined SOD activity, with GSH depletion in one hand and the increased MDA level on the other hand, in both the hippocampus and prefrontal cortex in response to restraint stress, which can be considered as an unsuccessful attempt of the antioxidant defense system in these regions, encountering the restraint stress-induced oxidative stress. Dietary broccoli consumption in rats resulted in the strengthening of the restraint stress-induced antioxidant defense system, as detected by enhanced GSH level and SOD activity in either region. In parallel, McWalter et al. have reported that steamed and cooked dietary broccoli consumption in rats demonstrated significantly enhanced induction of the survival signaling proteins including SOD1 and SOD2, documenting its cardioprotective properties against I/R-induced injury [10]. Upregulation of GSH level and downregulation of oxidative stress by broccoli sprout consumption have also been proved in the aorta, carotid, kidney, and heart of spontaneously hypertensive rats (SHRsp) [8], as well as in the liver of male Wistar rats [43].

It is noticeable that potentiating of antioxidant defense system was more remarkable in the hippocampus compared with prefrontal cortex. In prefrontal cortex, elevated level of GSH in the presence of broccoli compared with the stress group was still lower than its level in the control group. In addition, enhanced activity of SOD in the presence of broccoli in comparison with both the stress and control groups in hippocampus was more considerable than the pattern of SOD activity changes assessed in prefrontal cortex of three experimental groups. Consistently, while the potentiation of antioxidant defense system by broccoli in the hippocampus resulted in a reduction of MDA level in response to stress, such potentiation in prefrontal cortex was not strong enough to decrease the stress insult-induced MDA level, as it remained almost equal to the stressed condition. The antioxidant compounds in broccoli such as sulforaphane and kaempferol are proposed to either directly scavenge free radicals or indirectly increase endogenous cellular antioxidant defenses, for example, via activation of the Nrf-2 transcription factor pathway [5, 6, 44].

The Nrf-2 pathway is regarded as the most important in the cell to protect against oxidative stress [45]. The transcription factor Nrf-2 is the guardian of redox homeostasis. Under oxidant conditions, it activates a battery of antioxidant and cytoprotective genes that share in common a cis-acting enhancer sequence termed antioxidant response element (ARE), including heme oxygenase-1 (HO-1) and NQO-1 [46]. Enhancing expression of Nrf-2-derived genes protects the blood-brain barrier after brain injury [47], and activation of Nrf-2 pathway has also shown protection in the context of cognitive [48] and neurodegenerative disorders, such as AD and PD [49, 50], as well as against environmental toxicants [51].

Nrf-2 signaling pathway is in a close interrelation with another defensive pathway, mitochondrial biogenesis in cells [52]. Mitochondrial dysfunction is a pathological component of neurodegenerative diseases [53], as well as when cells are encountering various stressors [41]. Hence, an effective strategy for preventing and treating mitochondrial dysfunction-related diseases is the effective stimulation of mitochondrial biogenesis [54].

PGC-1*α*, NRF-1, and TFAM are mitochondrial pro-survival proteins involved in the mitochondrial biogenesis pathway. PGC-1*α* is activated under oxidative stress conditions, and in neuronal cells, it has been reported to be required for the induction of many ROS-detoxifying proteins, such as glutathione peroxidase (GPx), catalase, uncoupling protein 2 (UCP2), and superoxide dismutase 2 (SOD2) [55]. It is demonstrated that HO-1 regulates cardiac mitochondrial biogenesis via Nrf-2-mediated transcriptional control of NRF-1. Also, it is reported that Nrf-2 promotes the translocation of NRF-1 to the nucleus which in turn stimulates the transcription and translation of TFAM, which provokes transcription replication of the mitochondrial genome, promoting mitochondrial biogenesis [52].

In the present study, while, in the hippocampus, the expression of nuclear Nrf-2 and HO-1 in the Nrf-2 signaling pathway, and PGC-1*α*, and TFAM in the mitochondrial biogenesis pathway showed a significant elevation in response to stress compared with the control group, all the factors of protective Nrf-2 and mitochondrial biogenesis pathways behaved differently in prefrontal cortex and they all dropped in response to stress in comparison with the control groups. Interestingly, consumption of broccoli proved the potential to induce nuclear Nrf-2, HO-1, and NQO-1 in the Nrf-2 signaling pathway, as well as PGC-1*α*, NRF-1, and TFAM in the mitochondrial biogenesis pathway in both cerebral region and against the stress insult. However, such inducing potential was again stronger in the hippocampus than prefrontal cortex. Although these results are stated for the first time in the context of our study, mediation of protective effects of broccoli consumption and sulforaphane administration via induction of Nrf-2 signaling pathway has been reported previously by other investigations [10, 11, 43, 56, 57].

Neuroinflammation is a key element in the pathophysiology of neurodegenerative and stress-induced disorders [58, 59]. Significant enhancement of the two important inflammatory factors, TNF-*α* and nuclear NF-*κ*B in the hippocampus and prefrontal cortex in response to 6 hours of restraint-induced stress, confirms previous findings of other studies indicating induction of inflammatory response in brain against various types of stressors [41, 60–63]. The interesting finding of the present study was an even further significant induction of TNF-*α* and nuclear NF-*κ*B p65 against restraint stress in both cerebral regions of the broccoli receiving animals.

It is worth mentioning that the neuroinflammatory response is not always entirely detrimental and a dual role of inflammatory response has been evidenced [59]. For many years, it was thought that because NF-*κ*B is activated in cells under conditions where many cells die, NF-*κ*B plays a role in killing the cells. However, this interpretation flawed because it was based on “guilt-by-association” rather than on concrete data addressing cause-effect relationships [64]. Therefore, while today there are studies pointing to the detrimental role of inflammation and NF-*κ*B as well as TNF-*α* enhancement in various neuropathological conditions [65–67], there are several other studies pointing to the neuroprotective role of NF-*κ*B and TNF-*α* induction [64, 68–71]. As a whole, the overall neurotoxic/neuroprotective roles of TNF-*α* and NF-*κ*B in brain injury responses are complicated [64, 71] and will depend on several factors such as the extent of microglia activation in specific brain regions, timing, and threshold of their expression, on the conditions that stimulates their signaling [59]. For example, TNF-*α* released in the striatum has been proved to cause neurodegeneration, while its release in the hippocampus could promote neuroprotection [72]. TNF-*α* in neutrophils and epithelial cells stimulates injury, whereas in neurons it is neuroprotective [73]. NF-*κ*B activation by TNF-*α* in neurons is thought to be protective, but TNF-*α* and NF-*κ*B have induced production of inflammatory cytokines and neurotoxic substances in microglia [64, 74]. Taking all these reports into account, significant inductions of TNF-*α* and nuclear NF-*κ*B p65 in the hippocampus and in the presence of broccoli, besides other results obtained here regarding antioxidant defense system, as well as Nrf-2 and mitochondrial biogenesis-related factors in this group, support the protective potential of dietary broccoli consumption in the hippocampus of male rats against restraint stress-induced disturbances and at the time point of analyses.

Consistently, induction of apoptotic cell death proteins, cleaved form of caspase-3 and nuclear AIF, which were increased significantly in response to the stress insult, showed a considerable decline in the presence of broccoli and in the setting of hippocampus. In prefrontal cortex, the story was different though; significant elevation of TNF-*α* and nuclear NF-*κ*B p65 amounts, along with insufficient attempt of protective settings such as antioxidant defense system, as well as Nrf-2- and mitochondrial biogenesis-related factors against restraint-induced insult, have been associated with negative changes such as enhanced-MDA level and cleaved form of caspase-3 implying that presence of broccoli in the setting of prefrontal cortex has not been entirely protective, as observed in the hippocampus.

Such region-specific responses to an equal stressor and also broccoli pretreatment obtained here, are reasonable and in accordance with previous reports indicating that a similar stressor may differentially affect several brain regions. While repeated stress in the rat suppressed dentate gyrus neurogenesis and caused dendrites of hippocampus and medial prefrontal cortical neurons to shrink, it conversely caused basolateral amygdale neurons to increase in dendritic complexity and sprout new synapses [16]. Even differential behavioral neuronal subpopulation in a specific cerebral region has been documented. Chronic stress compromises the hippocampus in a region- and gender-specific manner, making only the CA3 region in male vulnerable to ibotenic acid neurotoxicity [75]. In addition, it is represented that phenotypically distinct neuronal subpopulations in the anterior subdivision of the rat basolateral amygdale are activated following acute and repeated stress [76].

The term “hormesis” has long been used to describe the phenomenon where a specific chemical is able to induce biologically opposite effects at different doses; most commonly there is a stimulatory or beneficial effect at low doses and an inhibitory or toxic effect at high doses [77]. Several heavily studied phytochemicals have been suggested to exhibit hormetic effects on cells with low doses activating signaling pathways that result in increased expression of genes encoding cytoprotective proteins including antioxidant enzymes and mitochondrial proteins [78]. For example, raw garlic homogenates have been reported to exert antioxidant potential in low doses, but its higher doses have been shown toxic to the heart, liver, and kidney [79]. Such hormetic effect has also been reported for sulforaphane in broccoli [78, 80].

Therefore, it may be suggested that the region-specific responses to the restraint-stress and broccoli consumption by rats at the present study could be attributed not only to the different basic metabolic conditions and differential availability of defensive and compensatory systems in the hippocampus and prefrontal cortex, but also to the possibility that resulting metabolites and active constituents of broccoli reaching to the hippocampus after its consumption may have been fallen within the protective low dose ranges, while it has not been the same for prefrontal cortex. Such assumptions remain to be elucidated using further examinations. Conduction of detailed dose-response studies for each individual phytochemical in broccoli, as well as the whole broccoli, will be crucial to establish the dose range in which hormesis pathways are activated in the human brain without the adverse effects that are likely at high doses [68]. Such dose-response and kinetic-oriented studies of dietary factors will lead to improvements in dietary interventions for disease prevention and treatment.

## 5. Conclusion

While, in diets, the whole broccoli is consumed, yet most data supporting the protective potential of broccoli against different cancers and several other diseases have focused on purified or semipurified sulforaphane, or a water extract of broccoli sprouts, rather than the whole broccoli. So there is also a need to design more experimental and clinical studies to evaluate the health effects of whole broccoli, specifically in the context of neuropathological conditions, to supplement the existing reporting on bioactive components and plant extracts.

## Figures and Tables

**Table 1:** Plasma corticosterone levels in the experimental groups.

Experimental groups	Plasma corticosterone level (ng/mL)
Control	268.973 ± 31.09
Stress	453.431 ± 29.954*
Treatment	366.169 ± 28.22^*¥*^

*N* = 6/group, One-way ANOVA; Tukey HSD; *P < 0.05: compared with control group; ^¥^ *P* < 0.05; compared with stress group.
